# Plasma markers of inflammation and hemostatic and endothelial activity in naturally overweight and obese dogs

**DOI:** 10.1186/s12917-016-0929-8

**Published:** 2017-01-06

**Authors:** R. Barić Rafaj, J. Kuleš, A. Marinculić, A. Tvarijonaviciute, J. Ceron, Ž. Mihaljević, A. Tumpa, V. Mrljak

**Affiliations:** 1 Department of Chemistry and Biochemistry, Faculty of Veterinary Medicine, University of Zagreb, Heinzelova 55, 10 000 Zagreb, Croatia; 2 ERA Chair team VetMedZg, Internal Diseases Clinic, Faculty of Veterinary Medicine, University of Zagreb, Heinzelova 55, 10 000 Zagreb, Croatia; 3 Department of Parasitology and Parasitic Diseases with Clinic, Faculty of Veterinary Medicine, University of Zagreb, Heinzelova 55, 10 000 Zagreb, Croatia; 4 Department of Animal Medicine and Surgery, Faculty of Veterinary Medicine, Regional Campus of International Excellence Campus Mare Nostrum, University of Murcia, Murcia, 30100 Espinardo Spain; 5 Veterinary Institute, Savska cesta 143, 10 000 Zagreb, Croatia; 6 Clinic for Internal Dieaases, Faculty of Veterinary Medicine, University of Zagreb, Heinzelova 55, 10 000 Zagreb, Croatia

**Keywords:** Canine, IL-6, HsCRP, Clotting factors, Platelets

## Abstract

**Background:**

Obesity is one of the most prevalent health problems in the canine population. While haemostatic parameters and markers of endothelial function have been evaluated in various disease conditions in dogs, there are no studies of these markers in canine obesity. This study was designed to evaluate the effect of naturally gained weight excess and obesity on inflammatory, hemostatic and endothelial biomarkers in dogs. A total of 37 overweight and obese dogs were compared with 28 normal weight dogs.

**Results:**

Overweight and obese dogs had significantly elevated concentrations of serum interleukin-6 (IL-6) and C-reactive protein (hsCRP). Number of platelets, activity of factor X and factor VII were significantly higher, while activated partial thromboplastine time (aPTT) and soluble plasminogen activator receptor (suPAR) were significantly decreased. Statistical analysis of high mobility group box – 1 protein (HMGB-1), soluble intercellular adhesive molecule -1 (sICAM-1) and plasminogen activator inhibitor type 1 (PAI-1) concentrations did not show significant differences between the total overweight and obese group and the normal weight group of dogs.

**Conclusions:**

Analytical changes in the dogs in our study reflects that weight excess in dogs can be associated with a chronic low degree of inflammation and a hypercoagulable state, where primary and secondary hemostasis are both affected. However obesity is not associated with impairment of endothelial function in dogs.

## Background

Obesity is a widespread health problem in dogs living in developed countries. The incidence of canine obesity is increasing in parallel with human obesity [1]. Current estimates indicate that more than 30% of dogs are overweight or obese, and canine obesity should be a serious concern both for veterinarians as well as for pet owners due to the high number of diseases that can be associated with this condition [1–3].

Although there are controversial results for dogs, a link between obesity and inflammation has been established in human obesity, and increases in interleukin-6 (IL-6) in obese humans were observed [4, 5]. Interleukin -6 is a pleiotropic cytokine with a wide range of biological activities in immune regulation, inflammation, and whole-body energy homeostasis [6–8]. One of the main effects of IL-6 is activation of hepatocytic receptors, resulting in increased synthesis of certain proteins [9]. This includes induction of hepatic C-reactive protein (CRP) and fibrinogen (FIB) production, which are known to be acute phase proteins in dogs [10] and major risk markers of cardiovascular complications in humans [11].

Inflammation and coagulation are closely linked, both in health and disease, and share common activation and regulation systems [12]. Various mediators that induce a chronic inflammatory state in obesity are closely connected with haemostatic disturbances and recent studies have shown that the obesity state is characterised by prothrombotic state in humans [13–15], rodents [16], cats [17] and pigs [18]. Obesity induces alterations of both intrinsic and extrinsic pathways increasing the activities of vitamin K-dependent clotting factors [15]. There is also evidences that primary hemostasis is affected by weight excess, characterised by increased platelet number in circulation in humans [14] and dogs [19]. In addition, impaired fibrinolysis due to increased concentrations of plasminogen activator inhibitor-1 (PAI-1), and the soluble form of plasminogen activator receptor (suPAR) could occur as a consequence of weight excess [20, 21]. While the mechanisms linking human obesity with prothrombotic changes, including upregulation of procoagulant factors, downregulation of anticoagulants and inhibition of fibrinolysis are beginning to be understood and explained [13, 15], haemostatic balance in canine overweight/obesity conditions remains uninvestigated.

Endothelial dysfunction is a complication described in human obesity and other species including mice [22, 23]. Disturbances in endothelial function can be assessed by two serum biomarkers, high mobility group box – 1 protein (HMGB – 1) and intercellular adhesive molecule -1 (ICAM - 1) [24, 25]. Fiuza et al. [26] demonstrated that HMGB-1 induces a proinflammatory change in human microvascular endothelial cells in vitro, being considered as a marker of endothelial function. ICAM -1 is also considered as a marker of endothelial activation [25] and is elevated in a broad array of disease states, including obesity [25]. Both biomarkers are increased in human obesity and are also related, since HMGB-1 induces a proinflammatory change in human microvascular endothelial cells in vitro, characterized by up-regulation of ICAM-1 and production of proinflammatory cytokines [26].

Haemostatic parameters and markers of endothelial function have been evaluated in numerous disease conditions in humans and mices, including obesity [25, 27], although there are no studies of these markers in canine weight excess. The aim of the present study was to establish whether naturally gained canine weight excess could produce changes in primary and secondary haemostasis as well as in markers of endothelial function.

## Methods

### Study population

This was a prospective study in which two groups were established: lean dogs and overweight and obese dogs. Clients from the University Veterinary Hospital were contacted and were asked whether they had dogs that would fulfill the criteria of admission to the study that consisted of being free of illness, and of vaccination or medication administration within 2 months prior to sample collection. In case of overweight and obese dogs, they should have been in this state for at least one year and not participated in a weight-loss program at the time of enrolment. If the dog fullfilled the inclusion criteria, its owner was asked to bring the dog to the hospital for a systematic examination, which included detailed clinical inspection and routine hematological and biochemical analysis.

All lean dogs (*n* = 28) were deemed healthy based on a detailed normal history, clinical inspection, hematological and biochemical laboratory results and that were all in the reference range. Mixed different breeds were represented. The lean group was matched in age and sex to the group of dogs with weight excess.

Each owner of overweight and obese (*n* = 41) dogs was questioned about the duration of weight excess. Various breeds were represented. The overweight and obese group of dogs consisted of 10 crossbreeds, 7 golden retrievers, 7 labradors, 2 pekingese, 2 belgian shepherds, 2 beagles, 1 bernese mountain dog, 1 pug-dog, 1 tornjak, 1 dalmatian dog, 1 great dane, 1 mexican hairless dog and 1 stafford. The normal weight group of dogs consisted of 7 crossbreeds, 6 golden retrievers, 4 labradors and 10 mixed breeds. In four overweight and obese dogs we determined two metabolic underlying conditions that could have been related to weight excess - hypothyroidism (2 dogs) and diabetes mellitus (2 dogs). These dogs were excluded from the study, so the total number of overweight and obese dogs was 37. The assessment of the nutritional condition was based on a 5-point body condition scoring (BCS) system [28]. A single investigator assigned the dog to either lean (BCS 3), overweight (BCS 4) or obese (BCS 5). In addition, the weight of the dogs was compared with the standard weight for the breed. The weight of overweight dogs was 15 – 30% above ideal and the weight of obese dogs was > 30% above ideal [29] Table 1.

**Table 1:** Characterization of the study population

	Normal weight	Overweight and obese
Number of dogs	28	37
Number of males and females (%)	40, 60	40, 60
Neutered/spayed	3	4
Age (years, range)	6.5 (2–14)	7.5 (2–15)
Weight excess (%, range)	0	28 (15–54)
Body condition score	3	4 and 5

Basic hematological and biochemical parameters of two groups of the dogs are shown in Table 2.

**Table 2:** Basic hematological and biochemical parameters in normal weight and overweight/obese dogs

Group	RBC	RDW	MCV	MCH	MCHC	HB	HTC
	(x10^12^/L)		(fl)	(pg)	(g/l)	(g/l)	(%)
N	6.0 5.3–8.5	14 11–16	72 65–77	23 20–26	328 309–369	162 135–193	49 38–58
O	7.0 5.5–9.2	14 11–16	71 60–76	23 21–31	329 179–457	170 121–233	50 37–62
Ref.	5.5–8.5	11–16	60–77	19–23	320–360	120–180	37–55
	WBC	NEU	MO	LY	EO	NS	Ba
	(x10^9^/L)	(%)	(%)	(%)	(%)	(%)	(%)
N	9.4 5.4–17.4	60 39–77	1 0–8	34 17–55	4 0–18	0 0–1	0 0
O	9.1 3.9–13.5	67 22–91	1 0–10	24* 5–71	3 0–23	0 0–1	0 0
Ref.	6–17	60–70	3–10	12–33	2–10	0–1	0–1
	BUN	CRE	BIL	GLUK	AST	ALT	YGT
	(mmol/l)	(μmol/l)	(μmol/l)	(mmol/l)	(IU/l,37 °C)	(IU/l,37 °C)	(IU/l,37 °C)
N	6.5 3.7–9.5	100 81–128	3.6 1.5–9.4	4.5 2.7–5.9	31 19–60	47 25–142	3 1–9
O	4.7* 3–12	93* 20–129	3.4 1.5–10.4	5.0 3.5–6.0	32 19–62	42 22–187	3 1–6
Ref.	3.3–8,3	44–140	1.7–8.6	3.6–6.5	−82	−88	−6

*RBC* red blood cells, *RDW* red distribution width, *MCV* mean cell volume, *MCH* mean cellular hemoglobin, *MCHC* mean cellular hemoglobin concentration, *HB* hemoglobin concentration, *HTC* hematocrit, *WBC* total leukocyte count, *NEU* segmented neutrophils, *MO* monocytes, *LY* lymphocytes, *EO* eosinophils, *NS* nonsegmented neutrophils, *Ba* basophils, *BUN* blood urea nitrogen, *CRE* creatinine, *GLUC* glucose, *BIL* total bilirubin, *AST* aspartate aminotransferase, *ALT* alanine aminotransferase, *YGT* gamma glutamyl transferase, *N* control normal weight dogs (*N* = 28), *O* total overweight and obese dogs (*N* = 37), median, minimum and maximum is shown, *ref*. reference range in our laboratory, *p ≤ 0.05

### Laboratory measurements

The dog owners were asked to fast their dogs for 12 h prior to presentation for blood sampling. A sample of 6 ml of peripheral venous blood was drawn into EDTA or citrate tubes (Becton Dickinson, Rutherford, NJ, USA). Plasma was separated from blood cells by centrifugation at 1200 g for 15 min. Two 0.5 ml aliquots of EDTA and citrate plasma were separated and transferred to -80 °C within 1 h of collection and stored until analysis. The hematological, biochemical and hormonal analysis were performed immediately after the venipuncture.

Fibrinogen concentration, clotting factor activity, prothrombine time (PT) and activated partial thromboplastine time (aPTT) were measured in citrate plasma by a coagulometric method, using ACL 7000 analyser (Instrumentation Laboratory, Bedford, USA) based on a canine calibration curve with original reagents from the manufacturer. We used a coagulometric assay with specific factor-depleted plasma to assess the biological activity of the measured clotting factors. The clotting factors FVII, FIX and FX were measured by procoagulant activity as F VII:C, F IX:C and F X:C in a coagulation end point assay using specific factor – depleted plasma, having less than 1% activity for the specific factor being measured and close to 100% activity of all other factors. The activity of the factors was determined relative to a canine pool of samples obtained from healthy animals and expressed as %. The calibration curve was made using the citrate plasma pool obtained from healthy animals. The intraassay coefficient of variation was less than 4%, while interassay precision was less than 7%.

Prothrombin time is dependent on the functional activity of clotting factors FVII, FX, FV, FII and fibrinogen. Platelet poor plasma was mixed with tissue factor at 37 °C and an excess of calcium chloride was added to initiate coagulation. The time taken from the addition of calcium to the formation of the fibrin clot is known as the PT. The aPTT measures the activity of the intrinsic and common pathways of coagulation. Platelet poor plasma was incubated at 37 °C and phospholipid and a contact activator were added followed by calcium. The aPTT is the time taken from the addition of calcium to the formation of a fibrin clot, expressed in seconds.

The number of platelets (PLT) and their mean volumen (MPV) were evaluated using an automatic blood cell counter "Horiba ABX" (Diagnostics, Montpellier, France) and original manufacturer's reagents.

High mobility group box – 1, sICAM-1, hsCRP, IL-6, PAI-1 and suPAR were determined using ELISA kits specific for canine samples manufactured by Biotang (Biotang Source International, Camarillo, USA) following the manufacturer's instructions. All of the measurements were performed at the same time in order to avoid procedural variations. All the species specific ELISA kits used had a similar basis. Purified canine specific antibodies against the analyte to be measured were pre-coated onto a microplate. The standards, controls and samples were added into the wells. After washing away any unbound substances, a HRP-labeled antibody was added to form a complex of antibody-antigen-enzyme labeled antibody. After a second washing to remove any unbound antibody- enzyme reagent, tetramethylbenzidine substrate was added, producing a blue color when catalyze by the HRP enzyme. The reaction was terminated by addition of a stop solution that changes the solution color from blue to yellow. Optical density was measured with a microplate reader at 450 nm (BioTek Instruments, Vermont, USA). A standard curve, prepared from five standard dilutions in duplicate, was used for calculating the concentration of analyte in the canine samples, expressed in μg/ml, ng/ml or pg/ml. The intraassay coefficients of variation were less than 10%, and the interassay coefficients of variation were less than 12% for all measured analytes. The lower limit of assay detection was 6 ng/ml for HMGB-1, 1.25 μg/ml for sICAM-1, 16 pg/ml for IL-6, 0.30 μg/ml for hsCRP, 2 ng/ml for PAI-a and 20 pg/ml for suPAR.

### Statistical analysis

Distribution of all variables was tested by the Kolmogorov-Smirnov test. Analytes with a normal distribution were expressed as mean and SD and analytes with non-normal distribution by median and the 25th and 75th percentile. Differences between lean and weight excess dogs were tested by either *t* test or Mann–Whitney *U* test. Multiple linear regression analysis was performed to evaluate correlations between analytes in normalweight and overweight/obese dogs. Statistical analyses were performed with computer software (Statistica for Windows, StatSoft Inc.), with the level of significance set at *p* < 0.05.

### Ethical approval

The study protocol was approved by the Ethics Committee for Animal Experimentation, Faculty of Veterinary Medicine, University of Zagreb, Croatia. All dog owners gave written informed consent before entering the study. The study complied with local and international laws for the use of animals in clinical research.

## Results

All lean, overweight and obese dogs had concentrations of plasma HMGB-1, sICAM-1, IL-6, hsCRP, PAI-1 and suPAR in the range of the ELISA assay detection. Overweight and obese dogs had significantly elevated IL-6 and hsCRP compared with normal weight dogs (261 pg/ml vs. 227 pg/ml, *p* = 0.001 for IL-6; 4.2 μg/ml vs. 3.7 μg/ml, *p* = 0.027 for hsCRP).

When hemostatic variables were studied, overweight and obese dogs showed significantly higher values of average PLT number, activity of factor X and factor VII (315x10^9^/L vs. 234 x10^9^/L, *p* = 0.001 for PLT; 115% vs. 104%, *p* = 0.007 for FX, 131% vs 109%, *p* = 0.054 for FVII) and significantly lower values of aPTT and suPAR compared with lean dogs (10 s vs. 11 s, *p* = 0.022 for aPTT, 1990 pg/ml vs. 2598 pg/ml, *p* = 0.002 for suPAR).

Statistical analysis of HMGB-1, sICAM-1, PAI-1 concentrations and MPV did not show significant differences between the total overweight and obese group and the lean dogs (Tables 2 and 3).

**Table 3:** Values of the inflammatory, hemostatic and endothelial biomarkers measured in this study in normal weight and overweight/obese dogs

Parameter/unit	Normal weight	Dogs	Overweight	And obese dogs	R and *P* -value
	Mean ± SEM	Median; CI (P 25–P 75)	Mean ± SEM	Median; CI (P 25–P 75)	
sICAM-1 μg/ml	320 ±13	321; 275–356	329 ± 8.2	326; 288–361	9.021; pd 0.536
IL–6 pg/ml	227 ± 8.9	220; 197–256	261 ± 4.9	259; 235–287	34.829; pd 0.001 **
HMGB-1 ng/ml	85 ± 4.5	83; 70–93	89 ± 2.6	89; 76–98	4.343; pd 0.384
hsCRP μg/ml	3.7 ± 0.17	3.7; 3.2–4.3	4.2 ± 0.14	3.9; 3.6–4.7	0.502; pd 0.027 *
suPAR pg/ml	2598 ± 136	2559; 2195–2923	1990 ± 126	1869; 1542–2486	−608.314; pd 0.002 *
PAI-1 ng/ml	70 ± 5.9	61; 44–94	75 ± 6.9	70; 43–86	1.145; np 0.611
FIB g/L	2.6 ± 0.16	2.6; 2.0–3.0	3.0 ± 0.20	2.6; 2.2–3.5	1.312; np 0.185
FIX %	96 ± 5.6	93; 77–106	112 ± 6.8	100; 84–140	1.385; np 0.081
FVII %	109 ± 6.2	110; 82–136	131 ± 8.7	125; 100–147	1.492; pd 0.054 *
FX %	104 ± 2.2	106; 94–112	115 ± 2.9	110; 102–128	11.557; pd 0.007 **
PT sec	6.1 ± 0.04	6.1; 6.0–6.2	6.1 ± 0.04	6.1 6.0–6.2	0.989; np 0.580
aPTT sec	11 ± 0.23	11; 10–12	10 ± 0.20	10; 9.3–11	−0.749; pd 0.022 *
MPV fl	8.3 ± 0.14	8; 8–9	8.6 ± 0.17	8; 8–9	0.318; pd 0.169
PLT x10^9^/L	234 ± 13	228; 201–265	315 ± 18	298; 253–401	80.916; pd 0.001 **

*HMGB-1* high mobility group box – 1 protein, *sICAM-1* soluble intercellular adhesive molecule -1, *IL-6* interleukin – 6, *hsCRP* high sensitivity C reactive protein, *PAI-1* plasminogen activator inhibitor type 1, *suPAR* soluble plasminogen activator receptor, *FIB* fibrinogen, *FX, FIX, FVII* clotting factors X, IX and VII, *PT* prothrombine time, *aPTT* activated partial thromboplastine time, *MPV* mean platelet volume, *PLT* number of platelets, *np* nonparametric distribution, *pd* parametric distribution

*p ≤ 0.05, **p < 0.01,

A significant positive correlation was found for IL-6 and HMGB-1 (*p* < 0.05, *r* = 0.62), and for FX and FIX (*p* < 0.05, *r* = 0.48), while a significant negative correlation was found for aPTT and FIX (*p* < 0.05, *r* = -0.51).

## Discussion

The connection between weight excess and inflammation, haemostasis and endothelial disturbances has mostly been explored in humans, as well as in mouse and rat models, while similar investigations are in their early stages in canine medicine.

Biomarkers of inflammation, IL-6 and hsCRP were increased in overweight and obese dogs (*p* = 0.001; *p* = 0.027). Although no changes in these inflammatory biomarkers have been detected after short-term experimentally induced obesity or weight loss [30], and some authors even reported decreased CRP in obese dogs [31], our results would be more in line with those obtained by German et al. [32], for obese dogs in a clinical setting. These values could reflect a chronic low degree of inflammation or even that relatively minor changes in CRP under reference ranges could reflect genetic, demographic, behavioural or dietary factors [33]. The primary source of circulating IL-6 in obesity could be macrophages that have infiltrated white adipose tissue and accumulated during obesity due to local hypoxia. One of the main effects of IL-6 is the induction of hepatic CRP and FIB production, playing a key role in the inflammatory processes associated with obesity [34, 35]. In addition, there is evidence that CRP is produced in the adipose tissue itself [36], so in addition to increased hepatic production, proliferating adipose tissue could also represent a source of this acute-phase marker in overweight and obese dogs.

Weight excess in dogs shifts the hemostatic balance and features a hypercoagulable state, which is characterised by increased activity of FX (*p* = 0.007) and FVII (*p* = 0.054), shortened aPTT (*p* = 0.022) and increased number of PLT in circulation (*p* = 0.001). Similar results indicating increased activity of vitamin K-dependent clotting factors were found in obesity in humans [37] and mices [38]. A connection between body fat content and altered haemostasis is also found in pigs [39]. An explanation could be increased liver production of clotting factor as a consequence of a chronic inflammatory state, but Cleuren et al. [38] found that in obesity the factors activity in plasma was not paralleled by changes in transcription levels in the liver. Moreover, Takahashi et al. [40] found that obese adipose tissue itself produced FVII, and that the production and secretion of FVII by adipocytes was enhanced by proinflammatory cytokines, so the possible role of chronic inflammatory state on canine adipocytes as an alternative source of some clotting factors remains to be investigated. A significantly shorter aPTT has been found in obese rats [16], humans [41] and mices [38], which is in line with our results. The clinical significance of short aPTT in diagnostics has recently gained interest, because aPTT might be usefull as a widely available and inexpensive marker of hypercoagulability [41].

The primary haemostasis in dogs with weight excess was affected due to increased platelet count, while the mean platelet volume remained unchanged compared with normal lean dogs. Similar findings have been found in obese dogs [19], pigs [18] and children [42]. Cytokine IL-6 was found to acts as a promotor of the maturation of megakaryocyte precursors, and this prothrombotic effect may explain the association between the increased markers of chronic inflammation and the elevated platelet count in obese humans [43], and possibly also in dogs.

In spite of the fact that obesity is characterized by decreased fibrinolysis in humans [13] where PAI-1 represents a part of the fibrinolytic system that is most disordered, in our experiments we did not find any changes in the PAI-1 concentration. In addition the soluble form of urokinase PAR, suPAR, was decreased in dogs with weight excess (*p* = 0.002). These findings disagree with other authors that investigated the effect of human obesity on the fibrinolytic system, where increased levels of suPAR was found [44, 45]. Further studies should be performed to determine the reason for these divergences in the behaviour of suPAR in canine and human obesity.

Proteins belonging to the HMGB-1 goup did not show a significant increase in dogs with weight excess. These proteins have been reported to increase in dogs with acute inflammation and neoplasms [46–49]. In our study we used species-specific canine antibodies and a highly sensitive assay for HMGB-1 that allowed us to quantify this protein in healthy control dogs, in contrast to other studies that reported no detectable concentration using assays designed for humans [47]. Our findings suggest that overweight condition and obesity are not associated with greater impairment of endothelial function in dogs. Similarly, HMGB-1 and sICAM-1 did not show significant increases in obese dogs in our study, contrary to what has been described in humans [50, 51]. One of the risk factors for atherosclerosis is an increase of sICAM-1. Therefore, the lack of increase of this protein in obese dogs could be related to the low frequency of atherosclerosis found in this species compared with humans, where it is a leading cause of mortality and is responsible for much of the morbidity [52].

Interleukin -6 was positively correlated with HMGB**-**1. A similar relationship between these two proteins was found by Zeng et al. [53] in human infectious disease. Moreover, Nativel et al. [54] concluded that HMGB**-**1 is an adipokine, which stimulates IL-6 secretion and may contribute to chronic inflammation in fat tissue of humans. Our finding suggest that HMGB-1 may play a role in the chronic inflammatory state in dogs.

The main study limitation was that client-owned animals living in a home environment were used, which contributed to study variability, where diet, exercise and husbandry were variable. In addition, we did not have the opportunity to follow the effect of weight loss on the measured biomarkers. Despite these limitations, this study provides a comprehensive analysis of proinflammatory events, haemostasis and endothelial function associated with weight excess in dogs. Further studies should be made in the future with a larger population of dogs to confirm these findings and in addition it would be interesting to compare in obese dogs whether the existence of obesity related metabolic symdrome (ORMD) could alter these analytes.

## Conclusions

In this report we showed that weight excess in dogs potentiates the prothrombotic state in apparently healthy animals. This could at least in part, be due to a chronic proinflammatory state. Altered haemostasis may be relevant as a pathological contributor to other haemostatic diseases if dog is overweight or obese. The fibrinolytic system in canine obesity and the role of decreased suPAR is not yet clear and needs to be further investigated. The positive correlation of IL-6 with HMGB-1 suggests that HMGB-1 could also have a role in the chronic inflammatory state in dogs.

## Abbreviations

ALT: Alanine aminotransferase
aPTT: Activated partial thromboplastine time
AST: Aspartate aminotransferase
Ba: Basophils
BCS: Body condition scoring system
BIL: Total bilirubin
BUN: Blood urea nitrogen
CRE: Creatinine
EO: Eosinophils
F VII:C, F IX:C and F X:C: Clotting factors coagulation end point assay
FIB: Fibrinogen
FX, FIX, FVII: Clotting factors X, IX and VII
GLUC: Glucose
HB: Hemoglobin concentration
HMGB-1: High mobility group box – 1 protein
hsCRP: High sensitivity C reactive protein
HTC: Hematocrit
IL-6: Interleukin – 6
LY: Lymphocytes
MCH: Mean cellular hemoglobin
MCHC: Mean cellular hemoglobin concentration
MCV: Mean cell volume
MO: Monocytes
MPV: Mean platelet volume
NEU: Segmented neutrophils
NS: Nonsegmented neutrophils
ORMD: Obesity related metabolic symdrome
PAI-1: Plasminogen activator inhibitor type 1
PLT: Number of platelets
PT: Prothrombine time
RBC: Red blood cells
RDW: Red distribution width
sICAM-1: Soluble intercellular adhesive molecule -1
suPAR: Soluble plasminogen activator receptor
WBC: Total leukocyte count
YGT: Gamma glutamyl transferase
